# Atypical Cause of Headache: A Potential Diagnostic Pitfall in Acute Medicine

**DOI:** 10.7759/cureus.108412

**Published:** 2026-05-07

**Authors:** Vasanthakumar Karumannan, Khadeeja Nauman, Sunil Zachariah

**Affiliations:** 1 General Internal Medicine, Surrey and Sussex Healthcare NHS Trust, Redhill, GBR

**Keywords:** acute angle-closure glaucoma, acute red eye, bilateral mydriasis, headache disorders, ophthalmology, peripheral iridotomy

## Abstract

Acute headache is a common clinical presentation with a broad differential diagnosis, ranging from benign to life-threatening conditions. Early recognition of atypical causes is essential, particularly when initial investigations are inconclusive. The etiology of acute headache can be broadly classified into primary and secondary causes. Primary headaches occur in the absence of identifiable structural pathology, whereas secondary headaches arise from an underlying organic cause.

In this case presentation, a 65-year-old woman presented with a severe headache, which was triggered by an episode of sneezing. The pain was severe, rated 10 out of 10 in intensity, described as a constant dull ache that was felt all over the head but was more prominent in the occipital region, and it was accompanied by photophobia. The pain was not relieved by pain medications and was not associated with fever or trauma. No past history of migraine. On examination, she had no neck stiffness or abnormal neurology. Eye examination revealed bilateral redness and dilated and fixed pupils. Pain continued to be severe despite regular analgesia. A CT head revealed no space-occupying lesion or bleed. She was admitted for close observation and consideration of further investigations, including MRI, lumbar puncture, and inpatient neurology review. Unfortunately, before the MRI, the headache worsened, and the patient also complained of reduced vision in both eyes. An urgent ophthalmology review was requested, which revealed increased intraocular pressure (IOP) in both eyes (right eye 39 mmHg, left eye 40 mmHg, normal IOP: 10-21 mmHg). She was diagnosed with bilateral acute angle-closure glaucoma (AACG). She was given medical management as per the glaucoma protocol; however, as the headache worsened, medical management failed to reduce ocular pressure. She was taken for emergency anterior chamber (AC) paracentesis due to refractory IOP and peripheral iridotomy on the same day as definitive management. Over the next few days, her vision and headache started to improve. She was discharged with regular follow-up with the glaucoma clinic.

## Introduction

Acute headache is a common and often diagnostically challenging presentation in the Emergency Department (ED). While most headaches are benign primary headache disorders, clinicians must rapidly identify secondary causes that may require urgent imaging, lumbar puncture, specialist review, or time-critical intervention. Red-flag clinical symptoms include thunderclap headache, new onset of headache after the age of 50 years, progressive symptoms, association with neurological signs and symptoms, fever, meningeal signs, visual disturbance, papilledema, and headache increased by exertion. The National Institute for Health and Care Excellence Clinical Knowledge Summary (NICE CKS) criteria provide a summary of how to identify the red flag symptoms and ensure patient safety to act faster while managing secondary causes of headaches [1]. 

The pathophysiology of acute headache varies according to the underlying cause. Primary headaches, such as migraine, tension-type headache, and cluster headache, arise from functional neurovascular, trigeminovascular, autonomic, or central pain processing mechanisms without an identifiable structural lesion. Gaul et al. state that migraine headaches arise from activation and sensitization of trigeminal nociceptors; essential processes are located at the meninges and the meningeal vessels [2]. In contrast, secondary headaches result from an underlying disorder, including intracranial hemorrhage, meningitis or encephalitis, cerebral venous sinus thrombosis, arterial dissection, raised intracranial pressure, giant cell arteritis, hypertensive emergency, sinus disease, or ocular emergencies. Hernandez et al. state that primary and secondary headaches are differentiated using clinical history, ranging from site, onset, pain score, aggravating and relieving factors, and associated symptoms [3]. 

Management of acute headache begins with a structured history and examination, including onset, severity, progression, triggers, associated neurological or systemic symptoms, medication history, and previous headache pattern. Examination should include neurological assessment, vital signs, fundoscopy where possible, and targeted eye examination when visual symptoms, red eye, abnormal pupils, or peri-orbital pain are present. Patients with red flags may require urgent CT brain, CT angiography, MRI/MR angiography or venography, lumbar puncture, inflammatory markers, or specialist referral depending on the suspected diagnosis. Conversely, typical primary headaches may be managed with appropriate analgesia, antiemetics, migraine-specific therapy, hydration, and avoidance of opioids where possible.

Acute angle-closure glaucoma (AACG) is an ophthalmological emergency that presents with a unilateral headache; however, the bilateral presentation is very rare, but it could happen. As mentioned in Khazaeni et al., the presentation is similar to a neurological emergency, and this happens due to an increase in the intraocular pressure (IOP) due to outflow obstruction of aqueous humor and, if untreated, can cause vision loss [4]. The incidence of angle-closure glaucoma is higher in the Asian population than in the White population due to a narrow anterior chamber (AC). In White individuals, it has been reported to be approximately two to four cases per year in 100,000 people in Europe, as per Chua et al. [5], as compared to six to 12 cases per year in 100,000 people in Asia, as per Zhou et al. [6]. AACG is more common in women than in men. The etiology is divided into primary and secondary. The major predisposing factor for primary is the structural anatomy of the AC. If the angle between the iris and cornea is shallow, patients have a higher risk of having AACG even with a transient IOP rise, such as the Valsalva maneuver. The secondary causes include pupillary block, non-pupillary block, drug-induced causes, and pushing mechanisms. The etiology in detail is explained in the discussion part of this article. AACG presents with severe unilateral eye pain and headache associated with blurred vision, nausea, vomiting, and rainbow-colored halos around bright light. This presentation could overlap with neurological presentation, and early detection will prevent blindness. This is the purpose of the article: to make readers aware that bilateral AACG is uncommon, and if present, it would present as a neurological presentation, and a delay in treatment would cause optic nerve damage. We emphasize the importance of differentiating the neurological and ocular emergencies and managing the condition according to clinical features, and approaching the presentation with a wider clinical approach. 

## Case presentation

A 65-year-old woman was brought to the ED by ambulance after developing a sudden-onset headache following a forceful sneeze and nose-blowing episode. She reported feeling unwell with cold-like symptoms before the onset. The headache initially began around the right eye and gradually spread across the frontal region. She described it as severe, sudden in onset, and unrelieved by painkillers. This headache was associated with blurred vision and photophobia; nausea with three episodes of vomiting overnight; three episodes of diarrhea, worsening with coughing and sneezing, and feeling unsteady when walking, and there was no neck stiffness. 

At presentation, her blood pressure was 199/79 mmHg, and the patient had a history of asthma, chronic obstructive pulmonary disease (COPD), and hypertension. The patient was diagnosed in the past with hypertension; however, she was inconsistent with her medication, ramipril, which was started by the GP. However, her subsequent blood pressure recordings of systolic blood pressure went below 150 mmHg. 

On examination, she was alert, conscious, and oriented, and was not able to see the lights; she kept her eyes closed. Glasgow Coma Scale (GCS) was 15/15. In terms of neurological examination, her power was 5/5 in all four limbs, there was no sensory deficit, and there were no cerebellar signs. The only abnormal finding was that both pupils were unreactive to light and dilated. Both eyes were red and watering.

**Figure 1:**
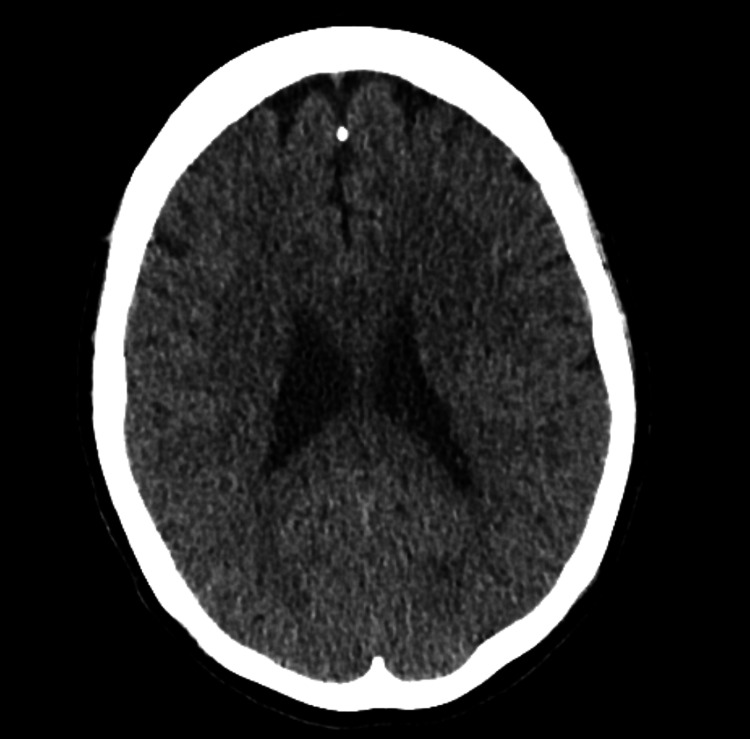
CT head The scan was taken within 12 hours of admission. Impression: No acute findings suggestive of subarachnoid hemorrhage (SAH), infarct, bleeding, or space-occupying lesions (SOL).

An urgent CT head did not show any space-occupying lesion or bleed (Figure 1). She was admitted for neurological observation and to consider further investigations, including MRI of the head, lumbar puncture, and inpatient neurology review. Her vision and headache started deteriorating, and an urgent inpatient ophthalmology review was requested. Baseline blood tests, including inflammatory markers, were normal.

During ophthalmology review, it was found that the patient had AACG, with increased IOP, right eye 39 mmHg, and left eye 40 mmHg (normal pressure 10-21 mmHg). As per the Royal College of Ophthalmologists' Glaucoma protocol, initially, she was prescribed timolol 0.5% eye drops, apraclonidine 1% eye drops, latanoprost 50 micrograms/ml 0.2 ml unit dose eye drops, and pilocarpine 2% eye drops. Oral acetazolamide was added to reduce fluid production. Despite both topical and oral tablets, the patient's symptoms continued to deteriorate. The ophthalmology team performed emergency AC paracentesis to reduce IOP and corneal edema, and following that, bilateral laser peripheral iridotomy was performed on the same day as definitive management. The rationale behind performing paracentesis was due to worsening vision, corneal edema, and refractory IOP despite medical management. Post-operatively, she was advised to continue with the following eye drops in both eyes: apraclonidine 1% (one drop three times a day for seven days), timolol 0.5% (one drop two times a day indefinitely), prednisolone 1% eye drops (six times a day for seven days), and pilocarpine 2% (one drop three times a day for seven days). However, prednisolone was changed to dexamethasone eye drops due to extensive inflammation. Eventually, the patient's IOP reduced to 8 and 4 mmHg in the right and left eyes, respectively (Table 1). 

**Table 1:** Right and left eyes' intraocular pressure monitoring and management from admission till discharge and follow-up Normal intraocular pressure: 10-21 mmHg. AC: anterior chamber; PI: peripheral iridotomy; IOP: intraocular pressure

Date and Time – Day 1	Medications Given at the Clinic - Right Eye	Right Eye IOP (mmHg)	Medications Given at the Clinic - Left Eye	Left Eye IOP (mmHg)
15:37	Timolol 0.5% eye drops – 1 drop	39	Timolol 0.5% eye drops – 1 drop	40
15:40	Apraclonidine 1% 0.25 ml unit dose eye drops preservative-free – 1 drop	39	Apraclonidine 1% 0.25 ml unit dose eye drops preservative-free – 1 drop	40
15:42	Latanoprost 0.005% eye drops – 1 drop	39	Latanoprost 0.005% eye drops – 1 drop	40
16:47	Pilocarpine 2% 0.5 ml unit dose eye drops – 1 drop	39	Pilocarpine 2% 0.5 ml unit dose eye drops – 1 drop	40
17:40	Acetazolamide 250 mg oral tablet – once only	39	Acetazolamide 250 mg oral tablet – once only	40
No response with medical management - emergency AC paracentesis on Day 1	Anterior chamber (AC) paracentesis was performed to have a clear cornea		Anterior chamber (AC) paracentesis was performed to have a clear cornea	
Later the same day – Day 1	Once the bilateral peripheral iridotomy (PI) was performed, the IOP was stabilized	13	Bilateral peripheral iridotomy (PI), the IOP was stabilized	8
Day 2 review after procedure: Due to inflammation, started on prednisolone; however, changed to dexamethasone due to extensive inflammation. Dexamethasone 0.1% 0.4 ml unit dose eye drops 1 drop every hour – 2 days 1 drop every two hours for 2 days 1 drop – six times a day for 7 days	Apraclonidine 1% 0.25 ml unit dose eye drops TDS – 1 drop pilocarpine 2% 0.5 ml unit dose eye drops TDS – 1 drop timolol 0.5% eye drops BD – 1 drop	8	Apraclonidine 1% 0.25 ml unit dose eye drops TDS – 1 drop pilocarpine 2% 0.5 ml unit dose eye drops TDS – 1 drop timolol 0.5% eye drops BD – 1 drop	4
Day 3 review	Apraclonidine 1% 0.25 ml unit dose eye drops TDS – 1 drop pilocarpine 2% 0.5 ml unit dose eye drops TDS – 1 drop timolol 0.5% eye drops BD – 1 drop dexamethasone 0.1% 0.4 ml unit dose eye drops 1 drop every hour – 2 days 1 drop every two hours for 2 days 1 drop – six times a day for 7 days	8	Apraclonidine 1% 0.25 ml unit dose eye drops TDS – 1 drop pilocarpine 2% 0.5 ml unit dose eye drops TDS – 1 drop timolol 0.5% eye drops BD – 1 drop dexamethasone 0.1% 0.4 ml unit dose eye drops 1 drop every hour – 2 days 1 drop every two hours for 2 days 1 drop – six times a day for 7 days	4
Day 14 review in the glaucoma clinic	Timolol 0.5% eye drops BD – 1 drop	7	Timolol 0.5% eye drops BD – 1 drop	8

The IOP came back to within normal limits. Tables 2, 3 summarize the for ocular pressure monitoring. In the follow-up glaucoma clinic appointment, the patient's IOP is being maintained within normal limits, and the peripheral iridotomy was intact. The patient is continuing timolol 0.5% eye drops and advised to follow up with the GP for blood pressure monitoring. 

**Table 2:** Ophthalmological examination (Day 1)

Examination	Right Eye	Left Eye
Conjunctiva/Sclera	Conjunctival hyperemia ++	Conjunctival hyperemia ++
Cornea	Corneal edema	Corneal edema
Anterior chamber	Bilateral angle-closure glaucoma	Bilateral angle-closure glaucoma
Pupil/Iris	Moderately dilated	Moderately dilated
Lens	2+ nuclear, 2+ cortical cataract	2+ nuclear, 2+ cortical cataract.

**Table 3:** Ophthalmological examination (Day 2)

Examination	Right Eye	Left Eye
Cornea	Corneal edema Descemet’s membrane folds	Corneal edema Descemet’s membrane folds
Anterior chamber	Deep anterior chamber AC flare 3+	Deep anterior chamber AC flare 3+
Pupil/Iris	Fixed dilated pupil patent peripheral iridotomy	Dilated pupil patent peripheral iridotomy
Lens	2+ nuclear cataract	2+ nuclear
Vitreoretinal	No fundal view – red reflex present	No fundal view – red reflex present

## Discussion

AACG is a time-critical ophthalmic emergency that can closely mimic life-threatening neurological conditions, leading to delays in accurate diagnosis.

The etiology of angle-closure glaucoma is classified into primary and secondary. The primary cause is mainly due to anatomical predisposition. The risk factors are the shallow AC, short axial length, increasing age, thickened lens, being female, and Asian descent. The mechanism is mainly due to pupillary block, which blocks the flow from the posterior to the AC, and the formation of iris bombe, which interrupts the aqueous humor flow. 

The secondary causes include pupillary block (uveitis, phacomorphic glaucoma, and lens subluxation); non-pupillary block causes (plateau iris syndrome, malignant glaucoma, and ciliary body edema), drug-induced causes (topiramate, sulfonamides, and selective serotonin reuptake inhibitor (SSRIs)); and pushing mechanisms from posterior to anterior (choroidal effusion and tumors), as mentioned in the International Classification of Headache in ED, by Friedman et al. [7]. In this case, the patient was not on any regular medications, except occasionally she took ramipril, which was for her hypertension. Other secondary causes were ruled out after complete ophthalmological evaluation. This suggests that, considering her age, being female, and the aggravating mechanism of sneezing and bilateral involvement of the eyes, it is due to the primary etiology of angle-closure glaucoma. Although AACG typically presents unilaterally, bilateral involvement can occur, particularly in anatomically predisposed eyes with shallow ACs or hyperopic features. As mentioned by Rafuse et al. and Renton et al., bilateral AACG may be precipitated by physiological stressors or Valsalva-type maneuvers such as sneezing, which can transiently increase IOP and narrow the iridocorneal angle further in at-risk individuals [8,9]. In this patient, we can predict that the etiology of the bilateral presentation is due to primary rather than secondary; however, the acute onset following a forceful sneeze may have contributed to angle-closure in both eyes, as mentioned in the literature. 

Filler et al. state that primary and secondary headaches have to be ruled out using available investigations to avoid any morbidity and mortality [10]. AACG can present only with headache, and at times, there is no ocular pathology at all, as discussed in Nesher et al. [11]. Early identification of the etiology would help the patient receive appropriate management.

This case highlights how AACG may initially present as an acute severe headache with systemic symptoms, resulting in early focus on neurological causes rather than ophthalmological evaluation. The patient's sudden, severe headache following a sneeze, associated with vomiting, photophobia, and elevated blood pressure, aligned with classical "red flag" features, prompting investigation for ophthalmological and neurological causes. The ophthalmological causes could be conjunctivitis, uveitis, episcleritis, carotid dissection, and glaucoma. Among these, due to no conjunctival edema, conjunctivitis was ruled out, and anterior uveitis is usually associated with a small pupil and an associated autoimmune disorder; this has been ruled out as well. Carotid dissection is usually presented as painful Horner's syndrome (ptosis, miosis, and neck pain) due to sympathetic chain involvement. The possibility of glaucoma stood out, and she was referred to ophthalmology to confirm the same. 

In terms of neurological differential diagnosis, there were no features suggestive of stroke, space-occupying lesion, infarct, bleeding, or injury. In terms of cluster headache, this usually presents unilaterally, and watering is one of the symptoms on only one side of the eye; it is a spectrum in trigeminal autonomic cephalgia. It occurs in clusters, and each episode lasts for minutes to hours. This is ruled out as well.

A CT scan was performed, which ruled out many of the neurological causes, and an MRI was booked to see any small infarct/bleeding as well. However, later, the ophthalmological intervention confirmed that this is AACG, and the MRI was cancelled. This neurologically focused pathway is well recognized as a standard diagnostic pitfall in AACG presentations. 

Headache as the predominant symptom is also recognized in AACG and can obscure underlying ocular pathology. Several studies, including Bhartiya et al., have reported that AACG may present with headache alone, without patients recognizing visual symptoms early in the course [12]. In this case, the patient initially described a severe occipital and frontal headache, an atypical pattern for AACG, but later reported progressive visual deterioration, photophobia, and inability to tolerate light. The presence of red eyes, watering, and fixed dilated pupils further supported an ocular rather than neurological cause.

Diagnostic overshadowing is common in emergency settings when a severe headache is prioritized as a neurological emergency. Although neuroimaging is necessary to exclude intracranial lesions, a routine CT scan should prompt reconsideration of the differential diagnosis before pursuing invasive procedures such as lumbar puncture. In this patient, the suggestion of a lumbar puncture was appropriate given the initial suspicion of CT-negative subarachnoid hemorrhage; however, the emergence of worsening visual symptoms mandated urgent ophthalmology review, which proved pivotal.

Prum et al. and Lam et al. emphasize that early recognition and management of AACG are essential to prevent irreversible optic nerve damage [13,14]. As per glaucoma guidelines mentioned in the Royal College of Ophthalmologists, standard first-line medical therapy includes IOP-lowering agents such as timolol, apraclonidine, pilocarpine, and systemic acetazolamide, which are used to reduce IOP. In cases where medical treatment is insufficient or if the medical treatment fails to reduce IOP as expected (refractory cases), urgent AC paracentesis is tried to reduce corneal edema and IOP, followed by bilateral peripheral iridotomy as a definitive management. In this patient, topical treatments were tried, followed by systemic acetazolamide, which did not provide results as expected. Due to corneal edema, urgent AC paracentesis followed by bilateral peripheral iridotomy was performed, which equalized the pressure between the anterior and posterior chambers and prevented recurrence. Friedman et al. emphasize the importance of including routine eye examination in headache work-ups, which will ensure patient safety [7]. 

## Conclusions

The learnings from this case report are that an acute headache with red eye presentation could be an ophthalmological emergency, rather than only a neurological complication. AACG can present as bilateral eye involvement, as opposed to the most common unilateral presentation. Bilateral AACG could damage the optic nerves and can become an ophthalmological emergency if not treated on time. This case emphasizes the importance of a broad diagnostic approach to acute headache in the ED. While neurological emergencies must be excluded, clinicians should maintain a high index of suspicion for ocular causes, especially when a patient presents with bilateral fixed or mid-dilated pupils; red eyes, watering, visual symptoms, and headache; or pain non-responsive to conventional analgesics. Incorporating routine ocular examination (pupil assessment, visual acuity, and inspection for conjunctival injection or corneal haze) into acute headache workup can substantially reduce the risk of misdiagnosis. In addition, involving the ophthalmological team in high-risk cases will ensure patient safety and avoid any potential complications, for instance, visual loss. 
